# Research and Application of Energy-Efficient Management Approach for Wireless Sensor Networks

**DOI:** 10.3390/s23031567

**Published:** 2023-02-01

**Authors:** Jinmeng Li, Jianxun Lv, Penghui Zhao, Yucheng Sun, Haiwen Yuan, Hai Xu

**Affiliations:** 1 School of Automation Engineering, Nanjing University of Aeronautics and Astronautics, Nanjing 210016, China; 2 Wuhu Machinery Factory, Wuhu 241000, China; 3 School of Automation Science and Electrical Engineering, Beihang University, Beijing 100191, China

**Keywords:** electric-field measurement, energy efficiency, high-voltage direct-current (HVDC) transmission, wireless sensor networks

## Abstract

Wireless sensor networks (WSNs) are widely used in industrial applications. However, many of them have limited lifetimes, which has been a considerable constraint on their widespread use. As a typical application of WSNs, distributed measurement of the electric field under high-voltage direct-current (HVDC) transmission lines also suffers from this issue. This paper first introduces the composition of the electric-field measurement system (EFMS) and its working principle. Considering the actual power supply of the system, this paper mainly introduces the composition of the wireless sensor node (WSND) and analyzes the power consumption and potential working state transformation of the WSND, together with a comprehensive study on parameters affecting the power consumption of the wireless communication unit. Moreover, an energy-efficient scheduling approach is proposed after specially designing a working sequence and the study on system parameters. The proposed approach is verified by experiments on not only the experimental line of the national HVDC test base, but also a commercial operation HVDC transmission line with the challenge of long endurance, which is considered in this paper with a new strategy. The results show that the proposed method can greatly extend the lifetime of the WSND.

## 1. Introduction

Wireless sensor networks (WSNs) have recently attracted extensive attention in both industry and academia. WSNs are widely used in distributed monitoring and control fields, such as environmental monitoring, industrial field control, smart homes, smart factories, and so on [1,2,3]. WSNs usually consist of a number of sensor nodes that complete a series of functions, such as information collection, processing, and transmission. Compared with traditional wired systems, a WSN has obvious advantages in terms of cost, flexibility, and convenience [4].

In many industrial applications, WSNs are powered by batteries, the limited capacity and inconvenient replacement requirement of which have become the main limitations on the application of WSNs. Lifetime power consumption management is of prime importance in WSN applications [5,6,7].

Energy-harvesting technologies, which harvest energy via different methods, have been developed in order to extend the effective lifetime of WSNs [8,9]. Baghaee et al. [10] present a WSN demonstration testbed powered by vibration energy. An electromagnetic harvester is designed to harvest vibration energy. In Ref. [11], researchers from Berkeley present an architecture for the perpetual operation of a WSN that utilizes environmental energy. The technology uses two-stage storage systems consisting of a supercapacitor and a rechargeable solar lithium battery. In Ref. [12], a human-energy-harvesting device with a power electronics module that can harvest energy generated by a walking human is designed and analyzed. Zhu Minglu et al. [13] make use of mechanical nanoenergy from the human body to investigate a self-powered physical sensor that exploits the potential of motion recognition and physiological signal monitoring. In Ref. [14], a cognitive industrial internet of things (CIIoT) with wireless energy harvesting is proposed, which harvests wireless energy from a primary user while performing spectrum sensing and transmissions. However, the energy-harvesting technologies in these studies require motion, such as mechanical vibration in the system, and also obtain less energy. Therefore, they have remained in the stages of laboratory design and verification and cannot be widely applied to industrial sites.

The main energy-consuming tasks in a WSN include data collection, transmission, and processing, which are also the main focus of energy consumption optimization. Nkwogu et al. [15] applied artificial neural networks to control data sampling to save node energy. Based on the spatiotemporal correlation of the sensor data, an adapting sampling method is proposed to extend the lifetime of the WSN [16]. Khan et al. [17] designed a cost-efficient radiation and temperature monitoring system using the ZigBee and Geiger Muller Tube. Taneja et al. [18] addressed an energy-efficient resource management model to select the optimal relay node and the optimal number of antennas at the mobile node to improve the system outage performance. Harb et al. [4] proposed an adaptive data collection mechanism to optimize data transmission and energy consumption. These methods can accurately manage energy, reduce energy consumption, and improve system lifetimes, and are mostly used in laboratory environments. However, there are a few cases in which these methods have been applied to complex outdoor conditions, such as electromagnetic measurement systems under HVDC transmission lines at present.

China has already started the commercial operation of several HVDC transmission lines. The electromagnetic environment is a factor that must be considered in the design, construction, and operation of transmission lines. The electric field under the HVDC transmission lines is an important indicator in electromagnetic environment evaluation [19,20,21]. A WSN-based electric-field measurement system (EFMS) is suitable for monitoring the electric field under the HVDC transmission lines. The electric-field sensor is the key part of the EFMS and often a point of focus in research. It has many implementation forms, such as MEMS, optical, and field mill sensors. MEMS sensors adopt the charge induction principle and use micromechanical resonators to convert the DC electric field into measurable electrical quantities [22,23]. Optical sensors adopt the principle of the electro-optic effect and use the influence of an electric field on the refractive index of an electro-optic crystal to measure the electric field [24]. Field mill sensors adopt the charge induction principle. The motor is used to drive the rotor to rotate at a constant speed, and the electric field is opened and shielded regularly, so that the induction electrode alternately induces the charge and disperses the charge to the ground, generating an AC signal proportional to the external DC electric field [19]. The motor inside the electric-field mill sensor needs to rotate continuously, which greatly increases the power consumption of the EFMS. MEMS and optical sensors negate the need for the motor and can reduce the power consumption of electric-field sensors [25]. However, due to cost and stability issues, most mature electric-field measurement systems currently use electric-field mills as electric-field sensors [26,27].

In order to study the electric-field distribution under the transmission lines, dozens of electric-field sensors are arrayed for uninterrupted measurement. However, in many test sites, there is no reliable and stable power supply, so EFMSs are usually powered by the battery. Due to the limited battery capacity in general and the system’s common requirements for data continuity, prolonging the effective lifetime of a battery-powered EFMS is critical but challenging. Moreover, with the rise in distributed sensor networks and real-time monitoring, the EFMS adds new functions, such as data communication [28], which further increases its energy consumption. The main goal of this paper is to establish an energy-efficient scheduling approach, which can be used to extend the lifetime of an EFMS. The paper is organized as follows. Section 2 presents the structure and composition of a wireless electric-field measurement system. Section 3 provides more details on the analysis of EFMS energy consumption, and the design of an energy-efficient scheduling approach. In Section 4, validation results for experimental lines in the national HVDC test base are provided together with those achieved for the actual HVDC transmission lines. This paper ends with a conclusion in Section 5.

## 2. Electric-Field Measurement System

The distribution of the total electric field can be obtained by measuring the electric field at different locations with multiple electric-field sensors scattered under the HVDC transmission lines [19].

**Figure 1:**
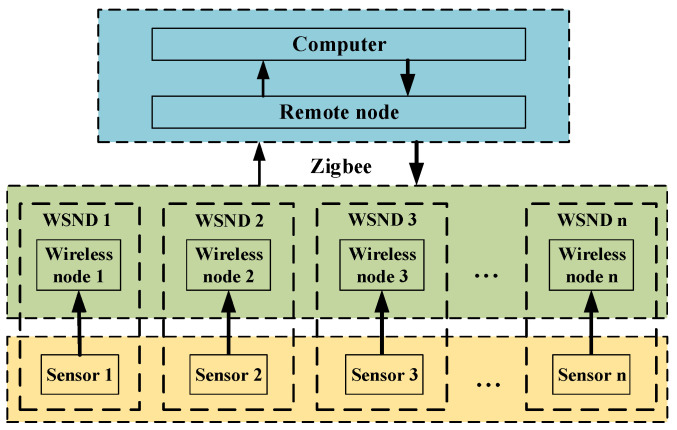
Structure of the wireless electric-field measurement system.

The framework of the EFMS is shown in Figure 1. The electric field measured by the electric-field sensor is periodically acquired by the wireless node and then transmitted to the remote node. The remote node transmits the data to the computer, and the computer establishes the electric-field distribution model under the HVDC transmission lines. The implementation of the EFMS was carried out according to our previous work [19,21]. Validation and experiments in the national HVDC test base have demonstrated that the proposed EFMS can accurately collect the electric-field data in a complex electromagnetic environment.

The computer is powered by commercial power or a small generator, and the remote node is powered by the connected computer. Meanwhile, the WSND is usually powered by batteries, especially in outdoor experiments. Therefore, the energy-efficient scheduling approach of the EFMS mainly focuses on the WSND.

**Figure 2:**
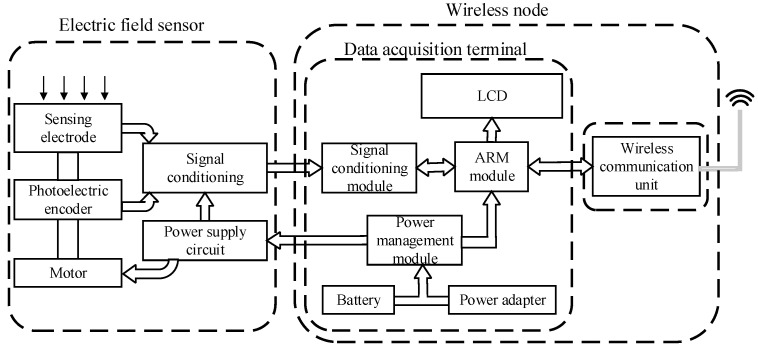
Schematic diagram of the wireless sensor node.

Figure 2 shows the architecture of the WSND. The WSND consists of an electric-field sensor (EFS), a wireless communication unit (WCU), and a data acquisition terminal (DAT) [21,29]. When the EFMS starts working, the motor in the EFS drives the rotor to rotate at a constant speed, and periodically switches on and shields the electric field. A sinusoidal induction signal is generated on the sensing electrode, and then the signal is converted from current to voltage through a special circuit. The DAT collects the voltage value and stores it in the internal ARM module for further processing. The processed signal is transmitted to the remote node through the WCU. During the operation of the EFMS, the WSND can be powered by batteries or power adapters according to the usage condition.

## 3. Energy-Efficient Scheduling Approach of WSND

In field experiments in the national HVDC test base or under actual ultra-high-voltage direct-current (UHVDC) transmission lines, we recorded the working time of each WSND. The battery-powered WSND had an effective lifetime between 14 and 19 h, averaging about 16 h. However, in order to understand the continuous change in the electric field over an entire day under the HVDC transmission lines, an operation time of 24 h is the goal. Therefore, we need to study the power consumption of the WSND and design an energy-efficient mode to extend its lifetime. Considering that the EFMS works periodically, we developed a sleep–wake strategy to reduce the average power consumption.

### 3.1. Overall Analysis of WSND Power Consumption

According to the composition of the WSND introduced in Section 2 and the main energy consumption source model of wireless sensor networks [30], the power consumption of each component was studied.

(1) Wireless Communication Unit

The WCU used in the system is Digi’s Xbee-Pro Radio Frequency Module, which adopts the Zigbee protocol (IEEE 802.15.4 standard). The basic performance and characteristics of the Xbee-Pro module are shown in Table 1.

It can be seen from Table 1 that the power consumption of the WCU varies greatly in different working states. The typical transmit current reaches 150 mA, while the receive/monitor current is only 55 mA.

(2) Data Acquisition Terminal

In the DAT, the unit with the largest power consumption is the ARM module [30,31]. The ARM module selected for the DAT was STM32F103VE. The datasheet shows that the ARM module has a low-power mode, and it takes about 55 μs to switch from IDLE mode to active mode. The consumption of the IDLE mode is only 10 μA. As ascertained in the field test, the current consumption of the DAT in active mode, measured by a series ammeter, is about 79 mA.

(3) Electric-Field Sensor

The motor inside the sensor is required to maintain a constant speed and cannot be stopped and restarted frequently to ensure the stability and accuracy of the EFS. Therefore, we set the EFS to work continuously. The current consumption of the sensor in the active state is about 40 mA.

By analyzing the power consumption and working status of different units of the WSND, we noticed that the following statements hold true:

(a) The main focus of energy management in the WSND is the WCU and DAT;
(b) The power consumption of the DAT is basically stable in active mode, and the ARM module is the main power consumption unit of the DAT;
(c) The power consumption of the WCU varies greatly with different operating conditions, such as transmit power.

### 3.2. Research on Characteristics of Wireless Communication Unit

We conducted a series of tests in the national HVDC test base to confirm the selection of transmit power levels and actual power consumption under different transmit powers.

The Xbee-Pro module supports five transmit power levels: 10 dBm, 12 dBm, 14 dBm, 16 dBm, and 18 dBm. The appropriate transmit power can be selected according to the communication distance and environment. The communication distance refers to the linear distance between the wireless node and the remote node. The environment refers to the weather conditions and pedestrian traffic around the equipment. “Simple” indicates that the equipment was placed on an open playground without passing pedestrians, “General” indicates that the equipment was placed on a remote road with occasional pedestrians passing through, and “Complex” indicates that the equipment was placed on a road with frequent pedestrians. Through the tests in the HVDC test base, the transmit power required for varying communication distances under different working environments and weather conditions was obtained, as shown in Table 2. The results show that, to ensure communication quality, the longer the distance and the more complex the environment, the bigger the transmit power required. Weather conditions also affect the quality of communication. Bad weather can lead to an increase in the required transmit power. However, to ensure the safety of the equipment, severe weather, such as heavy rain and thunderstorms, was avoided in the field test. Therefore, this paper does not consider severe weather conditions.

We also conducted experiments on the current consumption and data transmission time under different transmit power levels in the laboratory. In the experiment, the communication distance was set to 10 m. The environment was sunny, and no vehicles or pedestrians were passing around the equipment. The test results are shown in Table 3. “Monitor current” is the current consumed when the WCU was turned on but did not send data, and “Transmit current” is the current consumed when the WCU was turned on and sent data. We observed the following: (a) the monitor current under different transmit power levels is approximate; (b) the transmit current changed greatly as the transmit power increased; (c) for different transmit power levels, the data transmission time of the module was almost the same, at about 30 ms on average.

Based on the above two experiments, the transmit current of the WCU varies greatly under different transmit powers. To reduce the power consumption of the WCU, we set the transmit power level as low as possible. In the field test, the distances between the remote node and each WSND were between 10 m and 25 m. According to the test results in Table 2, we chose a transmit power of 10 dBm.

### 3.3. Energy Scheduling Method Design of WSND

**Figure 3:**
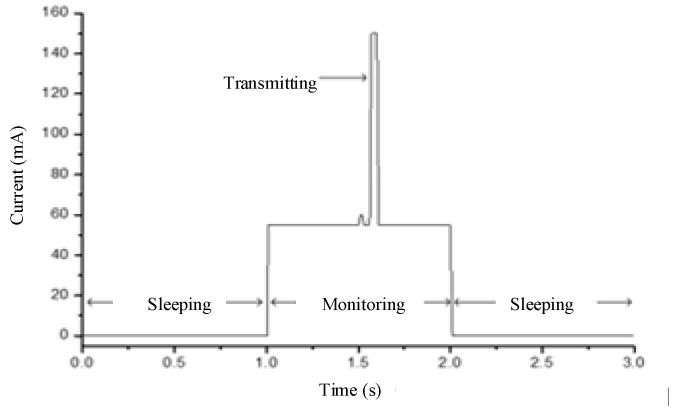
State transition diagram of Xbee-Pro module.

As mentioned above, the main objects to consider for energy management are the WCU and DAT. A state transition diagram of the WCU is shown in Figure 3. The DAT is either IDLE or active according to the operating mode of the ARM module. Therefore, the state of the WSND can be denoted as S0-S4, and the corresponding situations are shown in Table 4.

**Figure 4:**
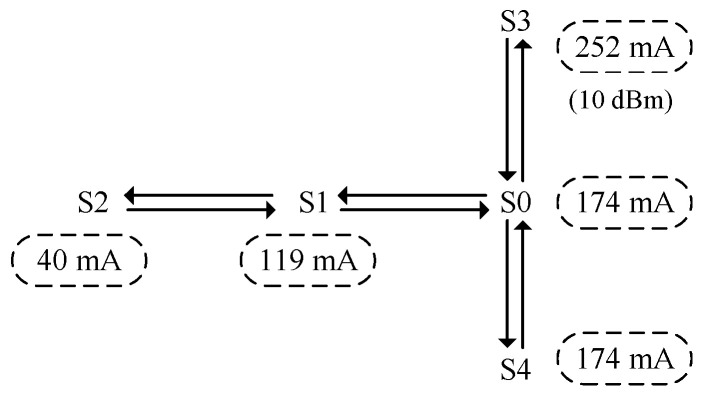
State transition and current consumption.

The state conversion of the WSND can be simplified, as shown in Figure 4. The figure also shows the total power consumption of the WSND in each state. As seen in Figure 4, there is a large gap in power consumption under different states.

**Figure 5:**
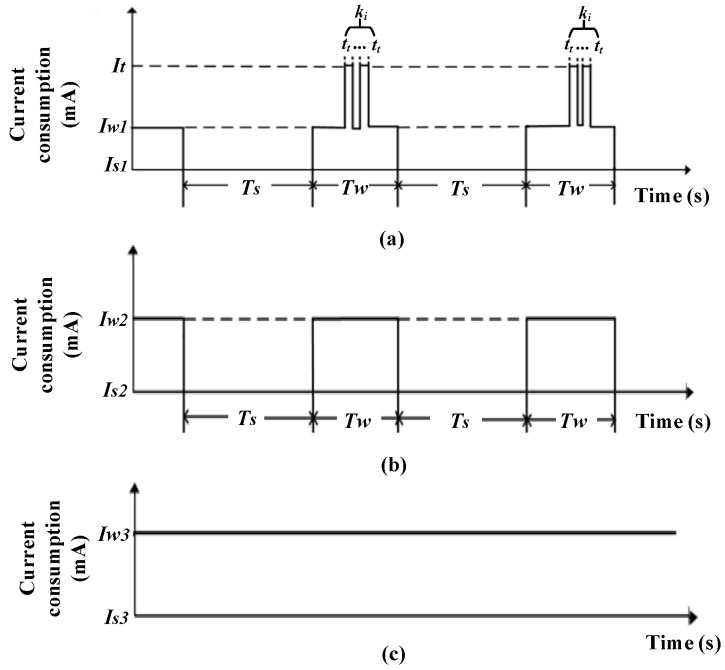
Working sequence diagram for (**a**) WCU, (**b**) DAT, and (**c**) EFS.

To reduce the average consumption and obtain a longer lifetime, a working sequence diagram for the three components of the WSND was designed, as shown in Figure 5. The device wakes up periodically and enters the IDLE state again after completing operations such as data processing, transmitting, receiving, etc. Since the state conversion times of the Xbee-Pro module and ARM module are very short, we ignored the corresponding conversion time. In Figure 5, *T_s_* represents the sleep time of all modules in each cycle. *T_w_* represents the active time of all modules in each cycle, *t_t_* represents the single data transmission time of the WCU, and *k_i_* indicates the total number of data retransmissions. *I_t_* represents the transmit current of the WCU. *I_w1_* represents the current consumption in the monitor state of the WCU. *I_w2_* represents the current consumption in the active state of the DAT. *I_w3_* represents the current consumption in the active state of the EFS. *I_s1_*, *I_s2_*, and *I_s3_* represent the current consumption of the WCU, DAT, and EFS in the IDLE state, respectively. The working cycle *T = T_s_ + T_w_*.

### 3.4. Lifetime Estimation of WSND

The electric-field measurement equipment uses 4 dry batteries with a voltage of 1.5 V or other rechargeable batteries to supply power, and each battery has a capacity of 2000 mAh. The operating voltage of the system is 3.3 V, and the theoretical equivalent system battery capacity is 3600 mAh.

#### 3.4.1. The Lifetime Calculation

As shown in Figure 5, the WCU and DAT are in the sleep–wake mode, and the EFS works continuously. The theoretical total energy consumption of the WSND can be calculated as follows:

(1) $$\begin{array}{l} Q_{all}=Q_{1}+Q_{2}+Q_{3}\\ =[I_{s1}\times T_{s}\times n+I_{w1}\times T_{w}\times n+(I_{t}-I_{w1})\times t_{t}\times n\times k_{i}]+\\ +(I_{s2}\times T_{s}\times n+I_{w2}\times T_{w}\times n)+(I_{w3}\times t) \end{array}$$

(2) $$n=\frac{t}{T_{s}+T_{w}}=\frac{t}{T}$$

where *t* is working time; *Q_1_* represents the energy consumption of the WCU; *Q_2_* represents the energy consumption of the EFS; *Q_3_* represents the energy consumption of the DAT; *n* is the total number of working cycles; and *k_i_* represents the number of data communications in the *i*-th working cycle.

To simplify the equation, we introduce the duty ratio *D*, which is described as follows:

(3) $$D_{}=\frac{T_{w}}{T}\times 100\%$$

At the same time, considering the sleep current of each module is very low, we set $I_{s1}=I_{s2}=0$. Thus, Equation (1) can be simplified as follows:

(4) $$\begin{array}{l} Q_{all}=I_{w1}\times t\times D_{}+(I_{t}-I_{w1})\times t_{t}\times n\times k_{i}\\ +I_{w2}\times t\times D_{}+I_{w3}\times t \end{array}$$

For comparison, the total energy consumption of the WSND without power management (D=1) is also given as follows:

(5) $$Q_{all-n}=I_{w1}\times t+(I_{t}-I_{w1})\times t_{t}\times n\times k_{i}+I_{w2}\times t+I_{w3}\times t$$

Furthermore, we obtained the average current consumption with or without an energy-efficient scheduling approach:

(6) $$I_{cc}=\frac{Q_{all}}{t}$$

(7) $$I_{\mathrm{cc}-n}=\frac{Q_{all-n}}{t}$$

The lifetime of the WSND with or without an energy-efficient scheduling approach can be calculated as

(8) $$\begin{array}{ll} T_{L} & =\frac{Q_{battery}}{I_{cc}}\\ & =\frac{Q_{battery}}{I_{w1}\times D+\left[(I_{t}-I_{w1})\times t_{t}\times n\times k_{i}\right]/t+I_{w2}\times D+I_{w3}} \end{array}$$

(9) $$\begin{array}{ll} T_{L-n} & =\frac{Q_{battery}}{I_{cc-n}}\\ & =\frac{Q_{battery}}{I_{w1}+\left[(I_{t}-I_{w1})\times t_{t}\times n\times k_{i}\right]/t+I_{w2}+I_{w3}} \end{array}$$

Based on the previous tests, the basic parameters for WSND lifetime calculation are shown in Table 5.

#### 3.4.2. Parameter Selection for the Energy-efficient Scheduling Approach

It can be seen from Table 3 that the XBee-Pro module takes about 30 ms to complete the communication process. Therefore, we use 30 ms to estimate the parameter of the system’s energy-efficient scheduling approach. If a node’s data transmission fails, the system will resend the data until the maximum time of data retransmission is reached. The working environment of the EFMS is relatively simple, so the maximum number of retransmissions for a single node is set to 5, and the maximum number of retransmissions for all nodes in each working cycle is 10. Beyond this, the host computer will set off an alarm and suggest troubleshooting.

Assuming that there are N WSNDs communicating with the remote node, the theoretical minimum communication time is 30 N ms. The retransmission interval is 26 ms. The total communication time *T_a_* in a working cycle can be calculated as follows:

(10) $$T_{a}=30N+\left(30+26\right)m_{j}$$

where *m_j_* represents the total number of retransmissions of all nodes in the *j*-th working cycle, *m_j_* ≤ 10.

Therefore, the maximum communication time of the EFMS for 1 working cycle is (30 N + 560) ms. When N = 10, the total communication time is less than 860 ms; when N = 20, the total communication time is less than 1160 ms. In order to provide some time margin for network communication to avoid network synchronization deviation caused by asynchronous sleep and clock offset, the non-sleep time and duty ratio can be set according to the number of nodes. For example, if the number of nodes ranges from 10 to 13, *T_w_* will be set to 1 s. If the number of nodes ranges from 14 to 17, *T_w_* will be set to 1.2 s. If the number of nodes ranges from 14 to 17, *T_w_* will be set to 1.3 s.

**Figure 6:**
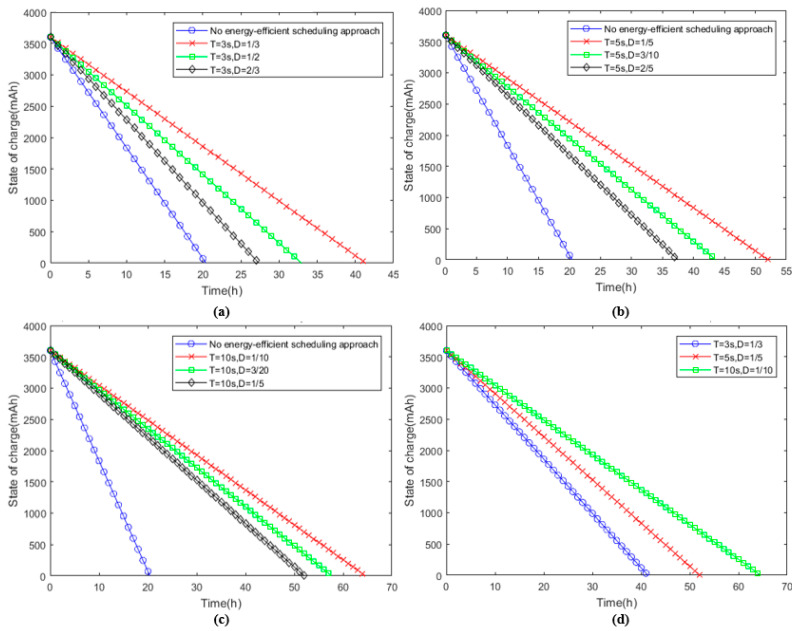
Comparison diagram of lifetime. (**a**) State of charge under different duty ratios when the period is 3 s. (**b**) State of charge under different duty ratios for 5 s. (**c**) State of charge under different duty ratios for 10 s. (**d**) State of charge with different cycles and the same wake-up time.

To study the impact of the duty ratio and cycle time on an energy-efficient scheduling approach, we simulated the change in the state of charge (SOC) of the WSND. The results are shown in Figure 6. For the theoretical calculation, we set *k_i_* = 1.

In Figure 6, the horizontal axis represents time and the vertical axis represents the state of charge. The initial capacity is 3600 mAh. It can be seen from Figure 6a–c that the duty cycle is the main factor affecting the lifetime of the WSND. Duty cycle and lifetime vary inversely. Therefore, when the cycle time is constant, the duty cycle should be set to be as small as possible. As seen in Figure 6d, when the active time is the same, the lifetime of the WSND increases with an increasing duty cycle.

## 4. Validation

**Figure 7:**
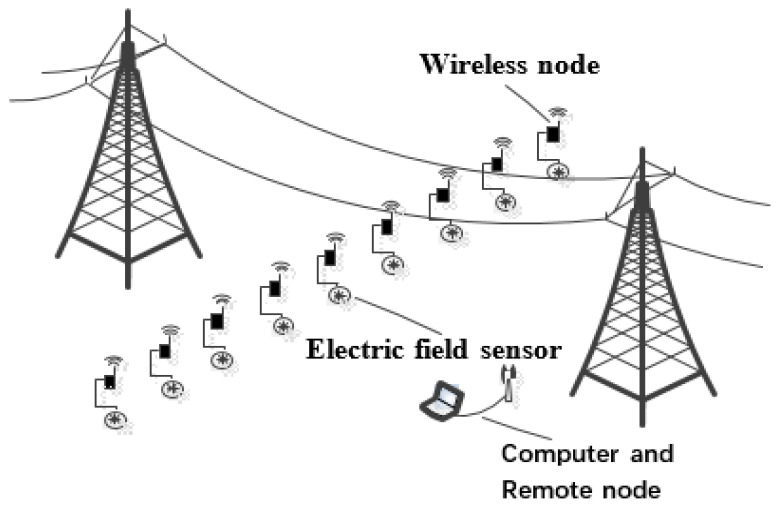
Arrangement diagram of electric-field measurement system.

We conducted tests on the experimental line of the national HVDC test base and actual UHVDC transmission lines to verify the effectiveness of the energy-efficient scheduling approach. As shown in Figure 7, the sensors and wireless nodes were arrayed linearly along the direction perpendicular to the transmission lines. According to the parameters of the transmission lines and test environment, 12 to 20 WSNDs were used in the experiments.

### 4.1. Test in the National HVDC Test Base

In the national HVDC test base, a 100 m long double-circuit experimental line, which consisted of a four-bundled conductor LJG-95/20, was used. The bundle spacing of the transmission line was 0.4 m. The minimum height of the conductors from the ground was 7 m, and the distance between the bipolar conductors was 6 m. Twelve WSNDs were set under the experimental line. The distance between any two adjacent WSNDs was about 3 m, and the maximum distance between the WSND and the remote node was about 30 m. There were no pedestrians passing around the equipment, that is, a “Simple” environment. The transmit power of the wireless nodes was set to 10 dB.

The active time was set to 1 s, and the monitoring period was set to 5 s. During the experiment, the highest temperature during the day was 28 ℃, the lowest temperature at night was 16 ℃, and the relative humidity fell between 34% and 65%. At the beginning of the test, all nodes were replaced with new batteries and then operated according to the proposed energy-efficient scheduling approach. The lifetimes of all WSNDs are recorded in Table 6.

### 4.2. Field Test under Actual UHVDC Transmission Lines

In the experiment, 12 WSNDs were placed linearly under the 1100 kV UHVDC transmission lines. The UHVDC transmission line adopted an eight-bundled conductor JL/G1A-1000/45. The bundle spacing of the transmission line was 0.45 m. The minimum ground height was 27 m, and the bipolar conductors were 26 m. The distance between two adjacent WSNDs was about 3 m, and the maximum distance between WSND and the remote node was about 25 m. Sometimes, pedestrians passed around the equipment, that is, a “General” environment. The transmit power of the wireless nodes was set to 10 dB.

The active time was set to 1.2 s, and the monitoring period was set to 5 s. The highest temperature during the daytime was 34 °C, the lowest temperature at night was 23 °C, and the relative humidity was between 29% and 45%. The wireless nodes’ batteries were replaced during the test’s beginning. The lifetimes of each WSND are shown in Table 7.

From Table 7, we can notice the following: (a) the lifetimes of WSND in this test were between 46.5 h and 47.2 h, and the fluctuation was slightly larger than that in the HVDC test base; (b) the average lifetime of the nodes was 46.88 h, slightly less than the theoretical value; (c) the nodes that were farther from the midline of the bipolar transmission lines than others had a shorter lifetime, but the change was not significant. The reason for this may be that nodes farther from the remote node are more susceptible to passing cars or other factors, so data retransmissions are more frequent.

Based on the two experiments in Section 4, we believe that the proposed energy-efficient scheduling mode can greatly prolong the lifetime of the WSND. Compared with the average lifetime of 16 h in previous onsite experiments, the proposed method can increase the lifetime to more than 46 h in the 5 s working cycle mode. This lifetime is nearly three times that of before and can meet the needs of experimental research.

## 5. Conclusions

In this paper, an energy-efficient scheduling problem for the EFMS was investigated. We proposed an energy-efficient scheduling method together with a corresponding parameter design principle. The validation results were achieved through an experiment conducted onsite. Both the experimental line of the national HVDC test base and an actual transmission line were utilized to validate the effectiveness of this method. Validation results show that the proposed method can effectively extend the lifetime of the battery-powered EFMS. In this paper, we presented two validation tests, in which the lifetime of all nodes of the EFMS reached an average of 50.71 and 46.88 h. Therefore, the proposed method can satisfy the needs of the study of electric-field distribution under HVDC transmission lines.

## Figures and Tables

**Table 1:** Basic specifications of Xbee-Pro Radio Frequency Module.

Parameter	Performance
Indoor range	100 m
Outdoor range	1500 m
Receiver sensitivity (1% PER)	−100 dBm
Data rate	250 kbps
Channels	16
Transmit current (typical)	150 mA
Receive/monitor current	55 mA
Power-down current	<10 μA
Maximum transmitted power	+18 dBm
Operating temperature	−40 °C to 85 °C

**Table 2:** Selection of transmit power levels.

	Environment	Sunny			Bad Weather (Rainy)		
Distance (m)		Simple	General	Complex	Simple	General	Complex
0–10		10 dBm	10 dBm	10 dBm	10 dBm	10 dBm	14 dBm
10–20		10 dBm	10 dBm	10 dBm	10 dBm	12 dBm	16 dBm
20–30		10 dBm	10 dBm	10 dBm	10 dBm	14 dBm	16 dBm
30–40		10 dBm	10 dBm	12 dBm	12 dBm	16 dBm	18 dBm
40–50		10 dBm	10 dBm	12 dBm	14 dBm	16 dBm	18 dBm
50–60		10 dBm	12 dBm	14 dBm	16 dBm	18 dBm	18 dBm

**Table 3:** Power consumption and transmission time under different transmit power levels.

Transmit Power Levels	Monitor Current (mA)	Transmit Current (mA)	Data Transmission Time (ms)
10 dBm	55	133	30.4
12 dBm	54	146	29.6
14 dBm	55	173	29.4
16 dBm	55	180	29.8
18 dBm	56	188	31.2

**Table 4:** States of wireless sensor node.

State	WCU	DAT	EFS
S0	Monitor	Active	Active
S1	IDLE	Active	Active
S2	IDLE	IDLE	Active
S3	Active (Transmit)	Active	Active
S4	Active (Receive)	Active	Active

**Table 5:** Basic parameters of node lifetime calculation.

$I_{si}$	$I_{w1}$	$I_{w2}$	$I_{w3}$	$t_{t}$
0	55 mA	79 mA	40 mA	30 ms
T	D	$T_{s}$	n	$I_{t}$
3 s, 5 s, 10 s	$T_{w}/T$	$T-T_{w}$	t/T	133 mA (10 dBm)

**Table 6:** Results of the WSND lifetime in HVDC test base.

No.	Lifetime (h)	No.	Lifetime (h)
1	50.7	7	50.8
2	50.6	8	50.9
3	50.7	9	50.7
4	50.5	10	50.8
5	50.8	11	50.8
6	50.6	12	50.7

**Table 7:** Results of the WSND lifetime measured under actual UHVDC transmission lines.

No.	Lifetime (h)	No.	Lifetime (h)
1	46.7	7	47.0
2	46.5	8	47.1
3	46.7	9	46.9
4	47.0	10	47.0
5	46.9	11	47.1
6	47.0	12	46.6

## Data Availability

The datasets generated and/or analyzed during the study are not publicly available but are available from the corresponding author on reasonable request.
